# Patterns and Risks of *Trichinella* Infection in Humans and Pigs in Northern Laos

**DOI:** 10.1371/journal.pntd.0003034

**Published:** 2014-07-31

**Authors:** James V. Conlan, Khamphouth Vongxay, Boualam Khamlome, Maria Angeles Gomez-Morales, Edoardo Pozio, Stuart D. Blacksell, Stanley Fenwick, R. C. A. Thompson

**Affiliations:** 1 School of Veterinary and Life Sciences, Murdoch University, Murdoch, Western Australia, Australia; 2 Division of Veterinary Services, Department of Livestock and Fisheries, Ministry of Agriculture and Forestry, Vientiane, Laos; 3 Department of Communicable Diseases Control, Ministry of Health, Vientiane, Laos; 4 Department of Infectious, Parasitic and Immunomediated Diseases, Istituto Superiore di Sanita, Rome, Italy; 5 Mahidol-Oxford Tropical Medicine Research Unit, Faculty of Tropical Medicine, Mahidol University, Bangkok, Thailand; 6 Centre for Tropical Medicine, Nuffield Department of Clinical Medicine, Churchill Hospital, Oxford, United Kingdom; National Institute of Parasitic Diseases, Chinese Center for Disease Control and Prevention, China

## Abstract

Several outbreaks of trichinellosis associated with the consumption of raw pork have occurred in Laos since 2004. This cross-sectional study was conducted in four provinces of northern Laos to investigate the seroepidemiology of trichinellosis in the human population and determine the prevalence and species of *Trichinella* infection in the domestic pig population. Serum samples and questionnaire data were obtained from 1419 individuals. Serum samples were tested for *Trichinella* antibodies by ELISA using larval excretory–secretory (ES) antigens and a subset of 68 positive samples were tested by western blot. The seroprevalence of *Trichinella* antibodies was 19.1% (95% confidence interval (CI) = 17.1–21.1%). The risk of having antibodies detected by ELISA using ES antigens increased with age, being of Lao-Tai ethnicity, living in Oudomxay province and being male. Tongue and diaphragm muscle samples were collected from 728 pigs and tested for *Trichinella* larvae by the artificial digestion method. *Trichinella* larvae were isolated from 15 pigs (2.1%) of which 13 were identified as *T. spiralis* by molecular typing; the species of the two remaining isolates could not be determined due to DNA degradation. *Trichinella* spp. are endemic in the domestic environment of northern Laos and targeted preventative health measures should be initiated to reduce the risk of further outbreaks occurring.

## Author Summary

Trichinellosis is one of the most widely distributed parasitic zoonoses worldwide and is caused by infection with nematodes of the genus *Trichinella*. Infection occurs after consuming larvae in the muscle of infected animals. Several outbreaks of trichinellosis have occurred in Laos since 2004, resulting in a substantial public health problem. The principal risk factor for trichinellosis is consumption of uncooked or partially cooked meat from domestic pigs and game. We visited communities in four ethnically diverse provinces of northern Laos to determine the seroprevalence of trichinellosis in the human population and explore the population and individual level risk factors of exposure. In addition, we also examined muscle samples collected from pigs post-slaughter to determine the prevalence of *Trichinella* infection and identify the species of *Trichinella* circulating in the domestic pig population. Our findings indicate that *Trichinella* spp. are endemic in the domestic environment of northern Laos and consumption of uncooked pork is common for all ethnic groups. Targeted preventative health measures, taking a one health approach by bringing together medical, veterinary and health sociology professionals, should be initiated to prevent the transmission of *Trichinella* and other foodborne pathogens in Laos.

## Introduction

Trichinellosis is one of the most widely distributed zoonoses worldwide and is caused by infection with nematodes of the genus *Trichinella* [1]. Infection occurs after consuming larvae in the muscle of infected animals with domestic and wild pigs the most common vehicles of human infections [2]. The severity of human disease is dependent on multiple factors including the number of viable larvae consumed, the frequency of consuming infected meat, meat being consumed raw or rare, the *Trichinella* species involved and individual susceptibility [3].

*Trichinella* spp. are endemic throughout Southeast Asia (SE Asia), from southern China to the Indonesian archipelago [4], [5] in domestic pigs and wildlife, causing frequent outbreaks of human disease. Three species of *Trichinella* have been detected in the SE Asian region, the encapsulated *T. spiralis* and the non-encapsulated *T. pseudospiralis* and *T. papuae* [6]. *Trichinella spiralis* has a regional distribution [7] with many of the recognised outbreaks occurring in the ethnically diverse regions of central and northern Laos, northern Thailand and northwest Vietnam where consumption of uncooked pork is common [8], [9], [10], [11]. Outbreaks of human trichinellosis involving *T. pseudospiralis* and *T. papuae* have occurred in Thailand after consuming wild pig meat [12], and cases of trichinellosis involving *T. papuae* have been detected in Papua New Guinea [15], [16] and a Thai patient returning from Malaysia [17].

Several outbreaks and sporadic cases of trichinellosis have occurred in Laos over the past five years [8], [18], [19] with the majority of the reported cases being associated with consumption of raw pork. Notwithstanding the propensity for Lao people to consume uncooked meat, including pork [20], little is known of the population and individual level risk factors of exposure and the meat consumption habits across an ethnically diverse country. Furthermore, relatively little is known about the prevalence of *Trichinella* infection in pigs and the species circulating in the domestic pig population. We report here the results of a cross-sectional serological survey of the human population and a concurrent survey in domestic pigs using muscle digestion in four provinces of northern Laos.

## Materials and Methods

### Ethics statement

Written informed consent was obtained from all participants 15 years and older and from the parents or legal guardians of children <15 years of age. The age of consent for this study was 15 years old. The study protocol was reviewed and approved by the Murdoch University Human Ethics Committee (Project no. 2008/266) and the Lao Ministry of Health National Ethics Committee for Health Research (no. 239/NECHR) before commencing this study. For the pig study, the protocol was reviewed and approved by the Murdoch University Animal Ethics Committee (Project no. R2108/07), which adheres to the Australian Code of Practice for the Care and Use of Animals for Scientific Purposes.

### Study sites

Laos is an ethnically diverse country with 49 distinct ethnic groups classified into four ethno-linguistic families (Lao-Tai, Mon-Khmer, Hmong-Mien, and Sino- Tibetan), comprising 67%, 24%, 8%, and 1% of the population, respectively [21]. The study was conducted in four provinces in northern Laos (Oudomxay, Luangprabang, Huaphan, and Xiengkhuang), where all four ethnolinguistic families are represented. One district in each province (Xay, Xiengngeun, Viengxay, and Pek Districts, respectively) was randomly selected for inclusion in this study.

### Human study design and risk factor questionnaire

The human survey was conducted in the dry season during January–March 2009 to maximize study participation and minimize negative impacts on seasonal labour demands. The survey design, sample size calculations and methodology have been described in detail elsewhere [20], [22]. The sample size calculations were based on estimates of taeniasis prevalence in the target populations. In brief, 14 households were randomly selected in each village and all household members ≥6 years of age were asked to participate. A venous blood sample of 2–3 mL was collected and the serum fraction was stored at −20°C. A household questionnaire was administered to the head of each household with his/her family present to assess the house characteristics, assets owned, ownership of animals, age of each household member, ethnicity and education levels, literacy of the male and female heads of household. Individual questionnaires were administered to collect data on meat consumption. For those family members who consumed raw meat, either pork, beef or fermented pork sausage, we asked them to estimate the frequency of raw meat consumption: weekly, monthly, every few months, and infrequently (once or twice per year or less often).

### Serological analysis and quality control

*Trichinella* excretory secretory antigens (ESA) prepared from *T. spiralis* larvae [23] and four positive control human serum samples were provided by the European Union Reference Laboratory for Parasites (EURLP; Department of Infectious, Parasitic and Immunomediated Diseases, Istituto Superiore di Sanita, Rome, Italy) and supplied lyophilised and stored at 4°C on receipt in Laos. In brief, *T. spiralis* muscle larvae were harvested from 3 month old CD1 female mice weighing 25 g which had been infected with 500 muscle larvae each 40 days before, by HCl–pepsin digestion and then maintained in culture for 18 h. Five hundred thousand larvae were washed three times for 20 min each time by sedimentation in a sterile 50 ml conical tube with 45 ml of warm sterile phosphate buffered saline (PBS), pH 7.3, supplemented with Penicillin and Streptomycin (25,000 mg/mL and 25,000 U/mL, respectively). At each change of the washing solution, larvae were gently shaken to dislodge adherent bacteria. The washing solution was removed after the final sedimentation of larvae and washed an additional five times by sedimentation in a sterile 50 ml conical tube with 45 ml of warm RPMI 1640 media supplemented with Penicillin and Streptomycin, as above. The larvae were resuspended in warm RPMI 1640 media supplemented with 5,000 mg/mL of Penicillin, 5,000 U/mL of Streptomycin, 200 mM Glutamine and 100 mM sodium pyruvate at a concentration of 5,000 larvae/ml and placed in 25 ml tissue culture flasks. The flasks were incubated in 5% CO2 at 37°C for 16–18 h. The larvae were separated from the medium by sedimentation in 50 ml conical tubes. The medium was filtered through a 0.2 µm filter and the larvae were discarded. The filtered medium was then concentrated (100x) in a pressure concentrating chamber using a YM-3 filter at 4°C and clarified by washing with PBS in the same chamber or by dialysing in PBS for at least 4 h. The protein concentration was checked by spectrophotometer at 260 nm and 280 nm, and each batch was aliquoted and lyophilized at 500 µg of total proteins per vial. The ESA and control sera were reconstituted in analytical grade water, aliquoted and stored at −20°C according to the manufacturer's instructions immediately prior to use. Reconstituted ES antigens were further diluted in carbonate buffered saline (pH 9.6) to a working concentration of 5 mg/ml and the reconstituted positive control sera were further diluted 1/200 in blocking solution (0.5% bovine serum albumin, 0.05% Tween 20 in PBS) for use in the assay. A panel of eight negative control serum samples were sourced from Lao people with no reported history of trichinellosis or of consuming raw pork. Negative control serum and test serum were diluted 1/200 in blocking solution for use in the assay.

The ES ELISA was performed in Laos at the National Centre for Laboratory and Epidemiology (NCLE) using a validated protocol [23], [24] with some minor modifications. Two positive control serum samples, 40 test serum samples and conjugate and substrate controls were added in duplicate to each plate; eight negative control serum samples were added to single wells of each plate. The optical density (OD) was measured at a wavelength of 450 nm using a microtiter plate reader (HumaReader, Germany). The cut-off on each plate was calculated as the mean OD of the eight negative control reference sera plus three standard deviations; a test ratio was calculated by dividing the OD of the test sample by the plate cut-off value and a test ratio ≥1 was considered reactive in the ES ELISA.

Sixty-eight samples that had an ES ELISA test ratio ≥1 were randomly selected from the pool of positive samples and sent to the EURLP for confirmatory testing. Samples were tested by the ES ELISA and western blot according to methods described elsewhere [23].

### Abattoir survey design

The abattoir survey design has been described elsewhere [20]. In brief, pig surveys were conducted at three slaughter-points in Xiengkhuang and Oudomxay Provinces from May–September 2008 and at two collection points in Huaphan and Luangprabang Provinces from October 2008–January 2009. The survey team consisted of trained district and provincial agricultural and forestry government staff who visited the slaughter points approximately every two weeks. The tongue and diaphragm pillar muscles were excised from all pigs brought for slaughter on the nights the survey team visited. Muscle samples were collected into labelled plastic containers and stored at 4°C before transport on ice to the National Animal Health Laboratory in Vientiane where samples were stored at 4°C prior to artificial muscle digestion.

### Artificial muscle digestion

Tongue and diaphragm muscle samples were artificially digested by the magnetic stirrer method in 1% pepsin (1∶10,000 US National Standard Formulary) and 1% hydrochloric acid (HCl) after removal of fat and fascia [25], [26]. Samples were tested in pools by muscle type with a maximum of 100 g per pool using 10 g of tissue per animal (if the animal was small >5 g of tissue was processed per animal). Tongue samples from positive pools were artificially digested as per the above protocol using 20 g muscle tissue (>10 g for small animals). Larvae were counted, transferred to 100% ethanol and sent to the EURLP for molecular species identification by multiplex PCR as previously described [27].

### Data analysis

The prevalence of human serum reactivity with *Trichinella* ES antigens was calculated for three diagnostic cut-offs in the ES ELISA, test ratios ≥1, ≥1.2 and ≥1.4. The level of agreement between the ES ELISA results from Laos and EURLP, and the level of agreement between the ES ELISA results from Laos and the western blot test were calculated for the three diagnostic cut-offs using the *Kappa* statistic. Sensitivity and specificity could not be calculated since no ES ELISA negative samples from Laos were subjected to further testing at the EURLP.

The questionnaire and laboratory test data were entered into a spreadsheet (Excel; Microsoft, USA) and subsequent analysis was carried out in STATA/IC version 10 (Stata Corp LP, USA). The socioeconomic status of each household was calculated by use of principal component analysis of household assets [28], [29] after replacement of missing values with the mean of the respective asset for that ethnic group. All assets were dichotomous. The households were ranked into wealth quintiles according to their cumulative standardized asset scores.

Univariate logistic regression without adjustment was used to test associations between ES ELISA reactivity and gender, location, ethnicity, age, wealth status and uncooked meat consumption habits. Risk factors significant or borderline significant (*P*≤0.20) in the univariate analyses were included in a multivariate random effects logistic regression model adjusting for the effect of household clustering. The results are reported as adjusted odds ratios and 95% confidence intervals (CIs). The final analysis only considered persons with serologic and questionnaire data.

In the pig study, the Pearson's chi-square test was used to explore associations between infection status (larvae detected by artificial digestion) and age, breed, sex and production system at last point of sale.

## Results

### Human study

A total of 1,582 persons in 332 households were eligible to participate in this survey. Of these persons, 1,419 (89.7%) individuals from 324 households aged 6–91 years provided a blood sample, a completed questionnaire, and had valid laboratory test results. The final survey population consisted of 583 Lao-Tai (93.6% compliance), 564 Mon-Khmer (95.4% compliance), and 272 Hmong-Mien (73.4% compliance). No Sino-Tibetan persons were recruited into this study. Survey population structures stratified by province are shown in Table 1. Significant differences in the survey population structure were observed for ethnicity and wealth status. Lao-Tai people made up the majority of the population surveyed in Huaphan province (95.6%), Mon-Khmer people made of the majority of the survey population in Oudomxay and Lauangprabang provinces (78.6% and 64.3%, respectively) and Hmong-Mien people made up the majority of the survey population in Xiengkhuang province (58.5%) (Table 1). Oudomxay province had the greatest proportion of participants who were *very poor* or *most poor* (68.5%) and this was reflected in the finding that Mon-Khmer people were the most impoverished ethnic group and Lao-Tai people were the least poor overall.

**Table 1:** Survey population structure, stratified by province, ethnicity, wealth status, age and gender.

	Total (%)	Oudomxay (%)	Luangprabang (%)	Huaphan (%)	Xiengkhuang (%)	χ^2^	*P*
**Gender**							
Female	719 (50.7)	217 (52.7)	187 (50.1)	141 (48.0)	174 (51.2)		
Male	700 (49.3)	195 (47.3)	186 (49.9)	153 (52.0)	166 (48.8)	1.6	0.659
**Ethnicity**							
Lao-Tai	583 (41.1)	59 (14.3)	102 (27.4)	281 (95.6)	141 (41.5)		
Mon-Khmer	564 (39.8)	324 (78.6)	240 (64.3)	0 (0.0)	0 (0.0)		
Hmong-Mien	272 (19.2)	29 (7.0)	31 (8.3)	13 (4.4)	199 (58.5)	>999.0	<0.001
**Wealth status**							
Most poor	253 (17.8)	123 (29.9)	55 (14.8)	17 (5.8)	58 (17.1)		
Very poor	270 (19.0)	159 (38.6)	32 (8.6)	36 (12.2)	43 (12.7)		
Poor	297 (20.9)	27 (6.6)	57 (15.3)	147 (50.0)	66 (19.4)		
Less poor	313 (22.1)	40 (9.7)	67 (18.6)	86 (29.3)	120 (35.3)		
Least poor	286 (20.2)	63 (15.3)	162 (43.4)	8 (2.72)	53 (15.6)	561.7	<0.001
**Age (years)**							
6–11	296 (20.9)	106 (25.7)	65 (17.4)	50 (17.0)	75 (22.1)		
12–19	329 (23.2)	93 (22.6)	92 (24.7)	66 (22.5)	78 (22.9)		
20–34	297 (20.9)	86 (20.9)	66 (17.7)	70 (23.8)	75 (22.1)		
35–49	277 (19.5)	76 (18.5)	83 (22.3)	55 (18.7)	63 (18.5)		
≥50	220 (15.5)	51 (12.4)	67 (18.0)	53 (18.0)	49 (14.4)	20.3	0.061

Sixty-eight samples with a test ratio ≥1 in the ES ELISA in Laos were tested at the EURLP by the ES ELISA method and all but one were confirmed positive, corresponding to 98.5% agreement. In comparison with the western blot test, only 35.3% (24/68) of the Lao samples had three diagnostic bands detected, a banding profile consistent with clinically confirmed trichinellosis (Figure S1) [23], [24]. Using a diagnostic cut-off test ratio ≥1.0, ≥1.2 and ≥1.4 for the ES ELISA, the level of agreement with the western blot test was 35.3%, 50.0% and 62.3%, respectively. The two-by-two tables comparing the western blot test results and ES ELISA at different diagnostic cut offs are presented in Table 2.

**Table 2:** *Trichinella* spp. western blot positivity verses ES ELISA positivity for diagnostic cut-off equal to standardised ratios of ≥1.0, ≥1.2 and ≥1.4 for 68 human samples.

	Western blot	
	−	+
**ES ELISA (≥1.0)**		
−	0	0
+	44	24
**ES ELISA (≥1.2)**		
−	15	5
+	29	19
**ES ELISA (≥1.4)**		
−	31	12
+	13	12

Using a diagnostic cut-off test ratio ≥1.0, ≥1.2 and ≥1.4, the prevalence of *Trichinella* antibodies detected by ES ELISA were 19.1%, 12.7% and 7.5%, respectively (Table 3). The prevalence of antibody detection by ES ELISA was highest in males, increased with increasing age to a peak in 35–49 year olds, increased with increasing wealth and was highest in the Lao-Tai ethnic group (Table 3). Prevalence was highest in Oudomxay province when a cut-off test ratio ≥1.2 and ≥1.40 were applied, and was highest in Xienghuang province when a cut-off test ratio ≥1.0 was applied (Table 3).

**Table 3:** Unadjusted prevalence of *Trichinella* ES ELISA positivity (95% CI) for diagnostic cut-off equal to standardised ratios of ≥1.0, ≥1.2 and ≥1.4.

		Proportion of human serum reactive in *Trichinella* ES ELISA		
	N (%)	Cut-off ratio ≥1.0	Cut-off ratio ≥1.2	Cut-off ratio ≥1.4
Total survey population	1419	19.1 (17.1, 21.1)	12.7 (11.0, 14.4)	7.5 (6.1, 8.9)
**Gender**				
Female	719 (50.7)	15.4 (12.8, 18.1)	11.0 (8.7, 13.3)	6.7 (4.8, 8.5)
Male	700 (49.3)	22.9 (19.7, 26.0)	14.4 (11.8, 17.0)	8.4 (6.4, 10.5)
**Province**				
Oudomxay	412 (29.0)	22.8 (18.8, 26.9)	17.2 (13.6, 20.9)	12.6 (9.4, 15.8)
Luangprabang	373 (26.3)	17.7 (13.8, 21.6)	11.0 (7.8, 14.2)	6.2 (3.7, 8.6)
Huaphan	294 (20.7)	11.2 (7.6, 14.8)	7.5 (4.5, 10.5)	3.7 (1.6, 5.9)
Xiengkhuang	340 (24.0)	22.9 (18.5, 27.4)	13.5 (9.9, 17.2)	6.2 (3.6, 8.7)
**Wealth status**				
Most poor	253 (17.8)	9.8 (6.2, 13.6)	5.5 (2.7, 8.4)	2.4 (0.5, 4.3)
Very poor	270 (19.0)	17.4 (12.9, 22.0)	12.2 (8.3, 16.1)	8.5 (5.2, 11.9)
Poor	297 (20.9)	14.8 (10.8, 18.9)	11.1 (7.5, 14.7)	5.7 (3.1, 8.4)
Less poor	313 (22.1)	24.0 (19.2, 28.7)	16.0 (11.9, 20.0)	9.6 (6.3, 12.9)
Least poor	286 (20.2)	28.0 (22.7, 33.2)	17.5 (13.1, 21.9)	10.8 (7.2, 14.5)
**Ethnicity**				
Lao-Tai	583 (41.1)	26.1 (22.5, 29.6)	19.2 (16.0, 22.4)	12.7 (10.0, 15.4)
Mon-Khmer	564 (39.8)	13.1 (10.3, 15.9)	7.6 (5.4, 9.8)	3.9 (2.3, 5.5)
Hmong-Mien	272 (19.2)	16.5 (12.1, 21.0)	9.2 (5.7, 12.6)	4.0 (1.7, 6.4)
**Age (years)**				
6–11	296 (20.9)	6.8 (3.9, 9.6)	4.1 (1.8, 6.3)	2.7 (0.9, 4.6)
12–19	329 (23.2)	12.1 (8.6, 15.7)	7.6 (4.7, 10.5)	3.3 (1.4, 5.3)
20–34	297 20.9)	22.6 (17.8, 27.3)	14.8 (10.8, 18.9)	8.4 (5.3, 11.6)
35–49	277 (19.5)	30.7 (25.2, 36.2)	20.6 (15.8, 25.4)	15.2 (10.9, 19.4)
≥50	220 (15.5)	26.8 (20.9, 32.7)	19.1 (13.9, 24.3)	9.5 (5.7, 13.4)
**Raw pork consumption**				
Does not eat	1224 (86.3)	18.0 (15.8, 20.1)	11.8 (6.0, 13.6)	7.0 (5.6, 8.5)
Weekly	20 (1.4)	15.0 (0.0, 31.1)	10.0 (0.0, 23.5)	5.0 (0.0, 14.8)
Monthly	66 (4.7)	18.2 (8.8, 27.6)	13.6 (5.2, 22.0)	7.6 (1.1, 14.0)
Every few months	71 (5.0)	31.0 (20.1, 41.8)	22.5 (12.7, 32.3)	15.5 (7.0, 24.0)
Infrequent	38 (2.7)	36.8 (21.3, 52.4)	23.7 (10.0, 37.4)	10.5 (0.6, 20.4)
**Raw beef consumption**				
Does not eat	917 (64.6)	14.7 (12.4, 17.0)	9.1 (7.2, 10.9)	4.6 (3.2, 5.9)
Weekly	52 (3.7)	30.8 (18.1, 43.4)	21.2 (9.9, 32.4)	13.5 (4.1, 22.8)
Monthly	205 (14.5)	29.8 (23.5, 36.0)	22.0 (16.3, 27.6)	17.2 (11.9, 22.2)
Every few months	168 (11.8)	24.4 (17.9, 30.9)	17.9 (12.0, 23.7)	9.5 (5.1, 14.0)
Infrequent	77 (5.4)	23.4 (13.8, 32.9)	14.3 (6.4, 22.1)	9.1 (2.6, 15.6)
**Raw fermented pork consumption**				
Does not eat	1023 (72.1)	15.0 (12.8, 17.1)	9.2 (7.4, 11.0)	5.3 (3.9, 6.7)
Weekly	96 (6.8)	25.0 (16.3, 33.7)	24.0 (15.4, 32.5)	13.5 (6.7, 20.4)
Monthly	149 (10.5)	33.6 (25.9, 41.2)	20.8 (14.3, 27.4)	14.1 (8.5, 19.7)
Every few months	104 (7.3)	33.7 (24.5, 42.8)	25.0 (16.6, 33.4)	16.3 (9.2, 23.5)
Infrequently	47 (3.3)	19.1 (7.8, 30.5)	12.8 (3.1, 22.4)	4.3 (0.0, 10.1)

**Figure 1:**
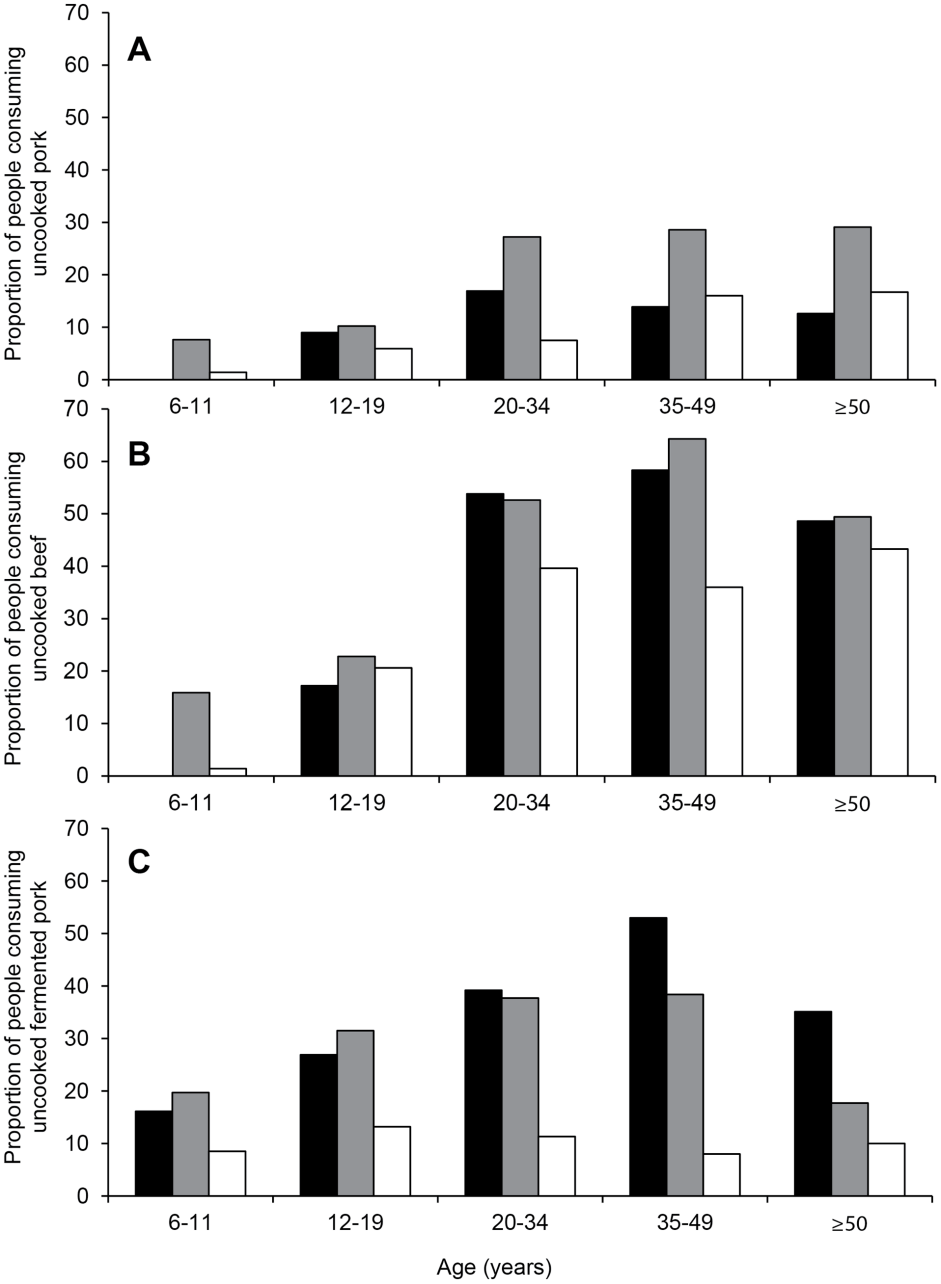
A-C, Proportion of the survey population reporting consumption of uncooked pork, uncooked beef and uncooked fermented pork, respectively, by age and ethnicity. Black columns, Lao-Tai ethnic group; Grey columns, Mon-Khmer ethnic group; and white columns, Hmong-Mien ethnic group.

The proportion of people reporting the consumption of uncooked beef, pork and fermented sausage peaked in older age groups for all ethnic groups (Figure 1) with the exception of Hmong-Mien people consuming fermented pork sausage, which was comparatively low for all age groups. The prevalence of antibody detection using a cut-off test ratio ≥1.0 was highest in people reporting consumption of raw pork (26.2% verses 18.0%), raw beef (27.1% verses 14.7%) and fermented pork sausage (29.8% verses 15.0%). Similarly, the prevalence of antibody detection using a cut-off test ratio ≥1.2 was highest in people reporting consumption of raw pork (18.5% verses 11.8%), raw beef (19.3% verses 9.1%) and fermented pork sausage (21.7% verses 9.2%). Using a cut-off test ratio ≥1.4, prevalence was highest in people reporting consumption of raw pork (10.8% verses 7.0%), raw beef (12.3% verses 4.6%) and fermented pork sausage (13.3% verses 5.2%). The prevalence of antibody detection stratified by frequency of raw meat consumption are summarised in Table 3.

**Figure 2:**
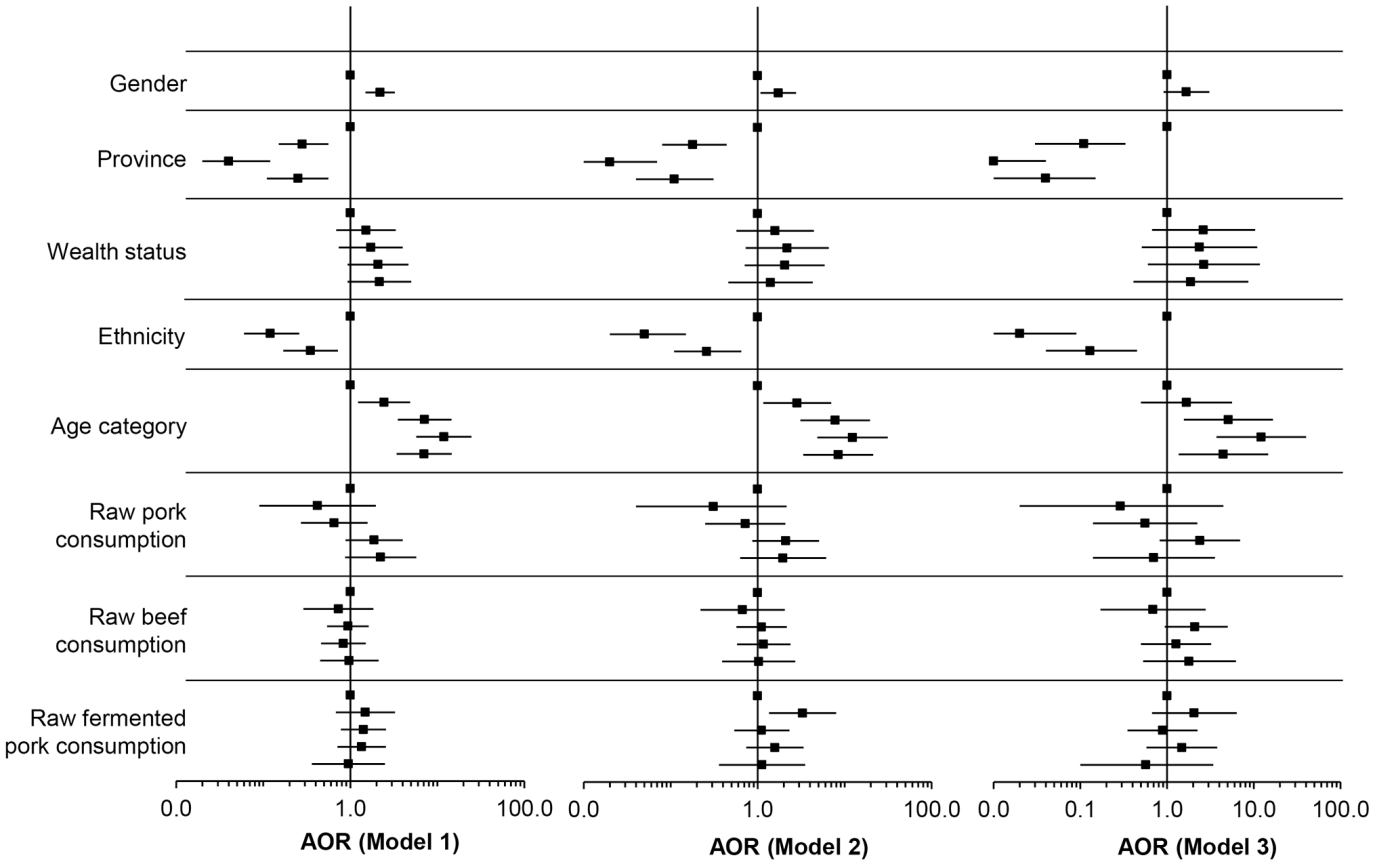
Adjusted odds ratio (AOR) of population characteristics associated with *Trichinella* ES ELISA reactivity, as determined by random effects multiple logistic regression modeling controlling for household clustering. All models adjusted for gender, province, wealth, ethnicity, age, frequency of raw pork consumption, raw beef consumption and fermented pork sausage consumption. Model 1; Diagnostic cut-off in *Trichinella* ES ELISA  =  standardised ratio ≥1.0; Model 2, Diagnostic cut-off in *Trichinella* ES ELISA  =  standardised ratio ≥1.2; Model 3, Diagnostic cut-off in *Trichinella* ES ELISA  =  standardised ratio ≥1.4. In descending order population characteristics are: Gender; female (referent), male. Province; Oudomxay (referent), Luangprabang, Huaphan, Xiengkhuang. Wealth status; most poor (referent), very poor, poor, less poor, least poor. Ethnicity; Lao-Tai (referent), Mon-Khmer, Hmong-Mien. Age category; 6–11 years (referent), 12–19 years, 20–34 years, 35–49 years, ≥50 years. Raw pork consumption; does not eat (referent), weekly, monthly, every few months, infrequent. Raw beef consumption; does not eat (referent), weekly, monthly, every few months, infrequent. Raw fermented pork consumption; does not eat (referent), weekly, monthly, every few months, infrequent (refer to data in Table S1).

After controlling for clustering at the household level, the risk of having *Trichinella* antibodies detected in the ES ELISA was significantly greater for people residing in Oudomxay province, people of Lao-Tai ethnicity, increasing age and being male. Increasing wealth was no longer associated with increased risk of having *Trichinella* antibodies detected after controlling for other risk factors (Figure 2). The frequency of consuming raw pork and beef were not associated with increased risk of having antibodies detected. Only consumption of fermented pork sausage on a weekly basis was significantly associated with increased risk of having antibodies detected when the diagnostic cut-off test ratio ≥1.2 (Odds ratio = 3.29 (95% CI = 1.35–7.99); Figure 2).

### Pig abattoir study

Tongue and diaphragm muscle samples were tested by the artificial digestion method from 728 pigs sampled from all four northern provinces included in the study. *Trichinella* larvae were isolated from 15 pigs (2.1%) of which 13 were identified as *T. spiralis* by molecular typing. Two isolates were not identified to the species level due to damaged DNA that may have occurred during tissue storage and muscle digestion. Prevalence of *Trichinella* spp. infection in pigs varied significantly (*P*<0.05) by province whereby the highest prevalence was recorded in Xiengkhuang (4.8%) and Oudomxay (2.8%) provinces (Table 4). No samples collected in Luangprabang province were infected with *Trichinella* larvae at the time of this survey. There was no significant difference in prevalence by breed, sex or the production system where the pigs were purchased immediately prior to slaughter.

**Table 4:** Prevalence of *Trichinella* spp. larvae isolated by artificial digestion from the tongue and diaphragm of pigs slaughtered at official slaughter points in four provinces of northern Laos.

	Prevalence of *Trichinella* spp. larvae detection
Animal production characteristic ¶	N (%)	% (95% CI)	*P*
Total survey population	728	2.1 (1.0, 3.1)	
**Province slaughtered**	728		
Oudomxay	144	2.8 (0.1, 5.5)	0.005
Luangprabang	209	0.0	
Huaphan	189	1.1 (0.0, 2.5)	
Xiengkhuang	186	4.8 (1.7, 7.9)	
**Age (months)**	656		
4–6	97	1.0 (0.0, 3.1)	0.282
7–12	3343	2.6 (0.9, 4.3)	
>12	216	0.9 (0.0, 2.2)	
**Breed**	666		
Indigenous	559	2.0 (0.8, 3.1)	0.691
Exotic	78	1.3 (0.0, 3.8)	
Cross-breed	29	0.0	
**Sex**	668		
Female	356	2.0 (0.5, 3.4)	0.532
Male	140	0.7 (0.0, 2.1)	
Castrated male	172	2.3 (0.0, 4.6)	
**Production system at last point of sale**	641		
Penned/corralled	464	2.4 (1.0, 3.8)	0.118
Free roaming	171	0.0	
Mixed	6	0.0	

¶ Indigenous and cross-breed pig breeds were from small backyard farms, even if from a penned system. The pig production system in northern Laos is quite complex and pigs raised in penned systems before going to slaughter may have been raised to weaner age in a free range system. The information on the production systems for the life of the pigs sampled could not be ascertained. Some of the exotic breeds were possibly produced in small intensive systems and at the time of the study, there were no large intensive piggeries in the provinces surveyed.

Of the 15 pigs infected with *Trichinella* larvae, 0.1–0.9 larvae per gram (lpg) of tongue tissue was detected in 10 pigs, 1–10 lpg was detected in three pigs and greater than 10 lpg was detected in two pigs. The highest recorded intensity of infection was 69 lpg in a pig slaughtered in Xiengkhuang province.

## Discussion

Trichinellosis is endemic in Southeast Asia with a concentration of outbreaks occurring in the ethnically diverse regions of northern Thailand, northern Vietnam and Laos [8], [9], [10], [11]. Our study confirms endemicity of *T. spiralis* in the pig population of Laos together with a spatial difference in prevalence of *T. spiralis* infection in pigs with worm burdens sufficient to cause severe human disease. One of the principle aims of the present study was to determine population and individual level risk factors associated with human exposure to *Trichinella* spp. larvae. Therefore we conducted a randomised cross-sectional survey of the human population in four northern provinces of Laos and used the ES ELISA as a serological measure of exposure. A high prevalence of *Trichinella* antibodies was detected by ES ELISA, with significant increased risk being associated with increasing age, Lao-Tai ethnicity, residing in Oudomxay province, being male and regular consumption of fermented pork sausage.

An important limitation of this study was the low participation rate of people from the Hmong-Mien ethnic group. The reasons for this low participation rate have been discussed elsewhere [20], [22]. Overall, the prevalence of ES ELISA reactivity across all ethnic groups increased with increasing age and prevalence was highest for males. In the Hmong-Mien ethnic group the ratio of females to males was similar for all age groups except the youngest group, where boys represented 60% of the age group. This discrepancy indicates that older males were over represented in the survey and our prevalence estimates are possibly higher than would otherwise have been the case if participation rates were higher. In addition, the highest non-participation rates in the Hmong-Mien group were observed in Huaphan province and this may have led to an over-estimation of prevalence of ES ELISA reactivity in this province.

No diagnostic test for trichinellosis, in any host species, has been validated for cross-sectional studies in Southeast Asia. The *Trichinella* ES ELISA lacks specificity owing to the relatively large population of antigens resulting in the detection of non-specific cross-reacting antibodies [23], [24]. The western blot test described by Gomez-Morales et al. [23], [24] was used as the gold-standard comparator for a small subset of ES ELISA positive samples in this study. The full spectrum of exposures, from subclinical infection, exposure to inactivated or injured larvae, old exposures through to acute and chronic clinical disease would lead to a varied serological spectrum at a population level. The ES ELISA results presented here are therefore imperfect but provide a measure of exposure at the population level.

Despite these limitations, we were able to demonstrate widespread serological evidence of exposure to *Trichinella* larvae in the human population. Increasing the diagnostic cut-off in the ES ELISA resulted in improved agreement with the western blot test, likely as a consequence of improved specificity at the expense of sensitivity. For this reason, prevalence was calculated for a range of diagnostic cut-offs in the ES ELISA to test the effect on the subsequent risk factor analysis. The pattern of risk for age, province of residence, gender, ethnicity and wealth status remained essentially the same as the diagnostic cut-off increased. For raw meat consumption, only the self-reported consumption of fermented pork sausage on a weekly basis was significantly associated with antibody detection in the ES ELISA at a cut-off test ratio ≥1.2. The consumption of fermented pork has previously been linked with an outbreak of trichinellosis in Bolikhamxay province in central Laos [18].

The lack of association with raw meat consumption, particularly raw pork, was somewhat unexpected since previously reported outbreaks of trichinellosis in Oudomxay and Bolikhamxay provinces have been linked with consumption of raw pork at festivals [8], [18]. This might be explained by the limitations of using single point-in-time self-reporting as opposed to asking the survey participants to keep a more detailed food diary. In general, methods of assessing dietary intake are imperfect and subject to error [30], especially in an ethnically diverse population with high rates of illiteracy and where Lao may have been the second language. From the data collected we were unable to estimate or correct for recall bias and the possibility that some survey participants may have misinterpreted the questionnaire cannot be ruled out. Future studies assessing risk associated with consuming uncooked pork should consider the use of a food diary to better estimate the prevalence of consuming uncooked meat.

In all risk models, the risk of having *Trichinella* antibodies detected by ES ELISA was highest in Oudomxay province compared to all other provinces. This finding may be an artefact of the large and widespread outbreak of trichinellosis that occurred in this province in 2005 [8] and be indicative of more widespread exposure over and above the clinical cases that were reported. More research, using more statistically powered surveys, is warranted in Oudomxay province to further investigate the risk of trichinellosis in this province.

The majority of pigs in which *T. spiralis* larvae were detected had a worm burden of less than 1 lpg and the infecting dose for clinically apparent trichinellosis has been estimated to range from ∼70-150 larvae [3]. The serological results together with the meat consumption habits and the abattoir survey results suggests that subclinical exposure may be common in Laos. Barennes and others (2008) reported apparently low morbidity associated with the 2005 outbreak in Oudomxay province and hypothesised that alcohol consumption may have diminished the severity of disease. Our results indicate that population immunity may have had a protective role.

The risk of *Trichinella* antibody detection in the ES ELISA increased significantly with increasing age and Lao-Tai people were at significantly greater risk. Regionally, trichinellosis has been associated with ethnically diverse mountainous areas of northern Vietnam and Thailand [9], [10], whereas in Laos we found the greatest risk associated with the majority lowland Lao-Tai population and the lowest risk was associated with people from the minority upland Mon-Khmer and Hmong-Mien ethnic groups. A high proportion of people from all ethnic groups reported consuming uncooked meat. Public health interventions, including a detailed assessment of the risks posed by ceremonial food preparation and the development of food safety education and awareness programs, could potentially reduce the transmission of *Trichinella* and other foodborne pathogens in Laos.

## Supporting Information

Checklist S1 STROBE checklist.
(DOCX)

Figure S1 Western blot (Wb) patterns of reactivity of excretory/secretory antigens (ESA) with human sera that tested positive by ELISA with ESA. M, molecular weight marker in kD; line 1, serum from a person with confirmed trichinellosis, positive control serum; line 2, Trichinella-specific proteins detected by Wb on an ELISA-positive serum from a Lao person; lines 3-5, non-diagnostic proteins recognized by Wb on ELISA-positive sera from three Lao people; line 6, ELISA negative control serum. The three-band pattern considered to be diagnostic is boxed in red.
(JPG)

Table S1 Adjusted odds ratio (AOR) of population characteristics associated with Trichinella ES-ELSA reactivity, as determined by random effects multiple logistic regression modelling controlling for household clustering.
(DOC)
