# Supplementary figures and images for: Patterns and Risks of Trichinella Infection in Humans and Pigs in Northern Laos
Source: PLoS Negl Trop Dis. 2014 Jul 31;8(7):e3034. doi: 10.1371/journal.pntd.0003034 (PMC4117436; doi:10.1371/journal.pntd.0003034)

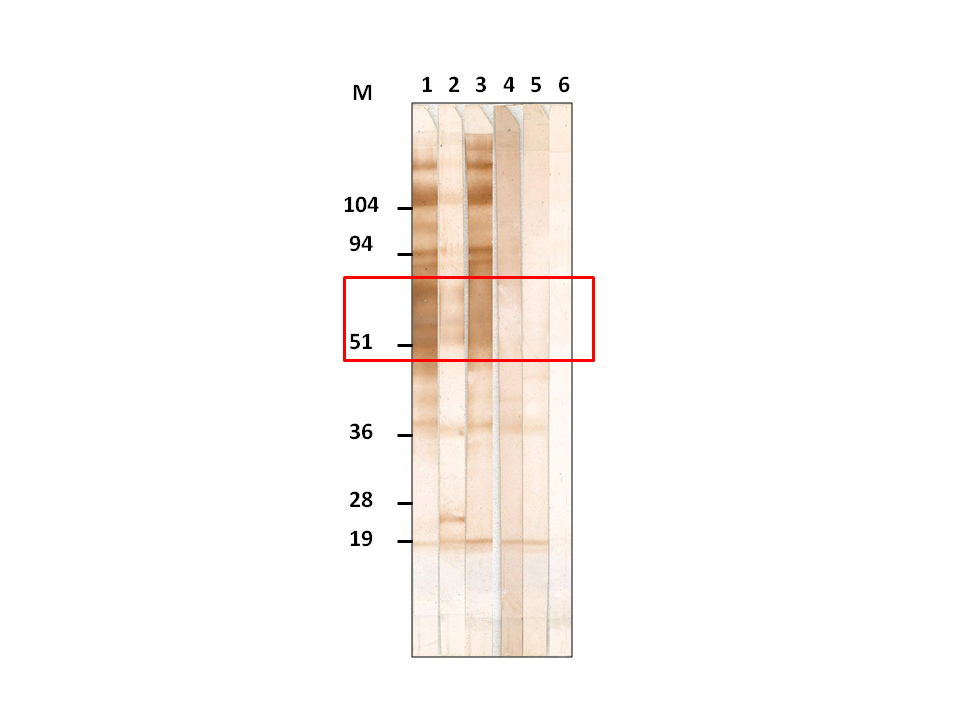

Supplement: Figure S1 — Western blot (Wb) patterns of reactivity of excretory/secretory antigens (ESA) with human sera that tested positive by ELISA with ESA. M, molecular weight marker in kD; line 1, serum from a person with confirmed trichinellosis, positive control serum; line 2, Trichinella-specific proteins detected by Wb on an ELISA-positive serum from a Lao person; lines 3-5, non-diagnostic proteins recognized by Wb on ELISA-positive sera from three Lao people; line 6, ELISA negative control serum. The three-band pattern considered to be diagnostic is boxed in red. (JPG) [file pntd.0003034.s002.jpg]
