# Supplementary material for: Patterns and Risks of Trichinella Infection in Humans and Pigs in Northern Laos
Source: PLoS Negl Trop Dis. 2014 Jul 31;8(7):e3034. doi: 10.1371/journal.pntd.0003034 (PMC4117436; doi:10.1371/journal.pntd.0003034)
Supplement: Table S1 — Adjusted odds ratio (AOR) of population characteristics associated with Trichinella ES-ELSA reactivity, as determined by random effects multiple logistic regression modelling controlling for household clustering. (DOC) [file pntd.0003034.s003.doc]

***Table S1*** *Adjusted odds ratio (AOR) of population characteristics associated with Trichinella ES-ELSA reactivity, as determined by random effects multiple logistic regression modelling controlling for household clustering.*

| **Population characteristic** | **Adjusted Odds Ratio^§^** | | |
| --- | --- | --- | --- |
|  | **Model 1^¶^** | **Model 2^¶¶^** | **Model 3^#^** |
| **Gender** |  |  |  |
| Female | Ref. | Ref. | Ref. |
| Male | 2.20 (1.49-3.24) | 1.73 (1.08-2.76) | 1.67 (0.91-3.08) |
| **Province** |  |  |  |
| Oudomxay | Ref. | Ref. | Ref. |
| Luangprabang | 0.28 (0.15-0.56) | 0.18 (0.08-0.44) | 0.11 (0.03-0.33) |
| Huaphan | 0.04 (0.02-0.12) | 0.02 (0.01-0.07) | 0.01 (0.00-0.04) |
| Xiengkhuang | 0.25 (0.11-0.56) | 0.11 (0.04-0.31) | 0.04 (0.01-0.15) |
| **Wealth status** |  |  |  |
| Most poor | Ref. | Ref. | Ref. |
| Very poor | 1.52 (0.69-3.33) | 1.59 (0.57-4.44) | 2.63 (0.67-10.37) |
| Poor | 1.72 (0.74-4.01) | 2.18 (0.73-6.55) | 2.36(0.51-10.97) |
| Less poor | 2.09 (0.93-4.67) | 2.05 (0.71-5.87) | 2.66 (0.60-11.71) |
| Least poor | 2.16 (0.93-5.01) | 1.41 (0.46-4.30) | 1.88 (0.41-8.64) |
| **Ethnicity** |  |  |  |
| Lao-Tai | Ref. | Ref. | Ref. |
| Mon-Khmer | 0.12 (0.06-0.26) | 0.05 (0.02-0.15) | 0.02 (0.01-0.09) |
| Hmong-Mien | 0.35 (0.17-0.72) | 0.26 (0.11-0.65) | 0.13 (0.04-0.45) |
| **Age (years)** |  |  |  |
| 6-11 | Ref. | Ref. | Ref. |
| 12-19 | 2.44 (1.23-4.86) | 2.85 (1.16-7.01) | 1.68 (0.5-5.60) |
| 20-34 | 7.15 (3.51-14.56) | 7.81 (3.11-19.62) | 5.10 (1.56-16.63) |
| 35-49 | 11.91 (5.75-24.67) | 12.32 (4.86-31.20) | 12.20 (3.71-40.09) |
| ≥50 | 7.06 (3.41-14.61) | 8.43 (3.35-21.26) | 4.45 (1.36-14.58) |
| **Raw pork consumption** |  |  |  |
| Does not eat | Ref. | Ref. | Ref. |
| Weekly | 0.42 (0.09-1.97) | 0.31 (0.04-2.15) | 0.29 (0.02-4.43) |
| Monthly | 0.65 (0.27-1.58) | 0.72 (0.25-2.08) | 0.56 (0.14-2.23) |
| Every few months | 1.88 (0.88-4.01) | 2.10 (0.87-5.07) | 2.39 (0.82-6.97) |
| Infrequent | 2.23 (0.87-5.75) | 1.96 (0.63-6.11) | 0.70 (0.14-3.56) |
| **Raw beef consumption** |  |  |  |
| Does not eat | Ref. | Ref. | Ref. |
| Weekly | 0.73 (0.29-1.84) | 0.67 (0.22-2.05) | 0.69 (0.17-2.78) |
| Monthly | 0.94 (0.54-1.63) | 1.11 (0.57-2.17) | 2.10 (0.94-4.99) |
| Every few months | 0.83 (0.46-1.50) | 1.17 (0.58-2.38) | 1.27 (0.50-3.22) |
| Infrequent | 0.97 (0.45-2.12) | 1.03 (0.39-2.69) | 1.80 (0.53-6.21) |
| **Raw fermented pork consumption** |  |  |  |
| Does not eat | Ref. | Ref. | Ref. |
| Weekly | 1.49 (0.68-3.26) | 3.29 (1.35-7.99) | 2.06 (0.67-6.36) |
| Monthly | 1.42 (0.78-2.57) | 1.11 (0.54-2.31) | 0.89 (0.35-2.25) |
| Every few months | 1.35 (0.71-2.56) | 1.58 (0.74-3.37) | 1.48 (0.58-3.78) |
| Infrequent | 0.95 (0.36-2.50) | 1.12 (0.36-3.51) | 0.57 (0.10-3.41) |

^§^ All models adjusted for gender, province, wealth, ethnicity, age, raw pork consumption, raw beef consumption and fermented pork sausage consumption;

^¶^ Diagnostic cut-off in *Trichinella* ES ELISA = standardised ratio ≥1.00;

^¶¶^ Diagnostic cut-off in *Trichinella* ES ELISA = standardised ratio ≥1.20;

^#^ Diagnostic cut-off in *Trichinella* ES ELISA = standardised ratio ≥1.40

Ref., referent variable.
